# Familiarity and Applications of Artificial Intelligence in Health Professions Education: Perspectives of Students in a Community-Oriented Medical School

**DOI:** 10.7759/cureus.73425

**Published:** 2024-11-11

**Authors:** Khaldoon Al-Roomi, Salman Alzayani, Amer Almarabheh, Mohamed Alqahtani, Fatmah Aldosari, Muneerah Aladwani, Noor Aldeyouli, Rahaf Alhobail, Hany Atwa, Abdelhalim Deifalla

**Affiliations:** 1 Department of Family and Community Medicine, Arabian Gulf University, Manama, BHR; 2 Department of Medical Education, Arabian Gulf University, Manama, BHR; 3 Department of Anatomy, Arabian Gulf University, Manama, BHR

**Keywords:** arab gulf, artificial intelligence, learning, medical education, medical student

## Abstract

Background and aim

Medical students are expected to be familiar with artificial intelligence (AI) applications in healthcare. This cross-sectional study looked at the attitudes, thoughts, and understanding of healthcare students toward AI.

Materials and methods

During the academic year 2023-2024, medical students enrolled in the College of Medicine and Health Sciences (CMHS) at the Arabian Gulf University (AGU) were included in this study. A questionnaire was developed to evaluate their understanding and opinions regarding the use of AI in medical training. These data were gathered, categorized, and analyzed using the Statistical Package for Social Sciences (SPSS) version 29 (IBM Corp., Armonk, NY, US). Categorical variables were shown in the form of frequencies and percentages, whereas continuous variables were presented as mean and standard deviation (SD). Chi-square tests were utilized for comparing categorical variables. A p-value of <0.05 was considered statistically significant.

Results

The study found that n=41 (27%) of medical students are very familiar with AI applications while n=92 (60.5%) are somewhat familiar. Familiarity increases as students progress in their medical education, with senior clinical phase students more familiar than juniors. There was no significant difference in perceptions of AI application among medical phases. Familiarity with research methodology and studies increases familiarity with AI applications. Most students believe AI will have a positive impact on medical education, but perceptions vary by educational phase. Many students support integrating AI into curricula with 67 (44.1%) of students using AI applications, with a higher percentage in pre-clinical phases, likely due to application in research projects in this phase. Concerns were raised about AI impacting the human touch in medical practice and doctor-patient communication, as well as technical challenges faced by students when applying AI.

Conclusion

Arab Gulf medical students show positive attitudes toward AI applications in medical education. Tailored educational strategies are needed to optimize AI integration in medical practice and address concerns effectively.

## Introduction

Artificial intelligence has attracted major attention during the past 10 years in view of its ability to process and solve health problems [1,2]. This came as a result of the increased utilization of machine-based and computer systems in most human-related fields, particularly medicine [3]. The integration of artificial intelligence (AI) into various sectors has revolutionized conventional practices, and in the healthcare sector, medical education is no exception and will soon facilitate wide-scale changes in this field. As medical schools strive to enhance educational outcomes and clinical competencies, it would be appropriate to conduct research initiatives to understand the familiarity and perceptions of health professions students regarding AI utilization.

Given the growing prevalence of AI technologies and their adoption in healthcare, many medical students now possess a foundational understanding of these tools, often acquired through coursework, workshops, and exposure to AI-driven applications in advanced conferences. The utilization of language-model AI or chatbots, particularly ChatGPT, is now an emerging trend that is welcomed by medical students and professionals in their daily activities. Notably, their familiarity with AI can significantly influence their acceptance of these technologies, as integral components of their inevitable future practice. Positive attitudes toward AI applications can enhance their learning experiences, enabling personalized education, improving diagnostic capabilities, and ultimately leading to better patient outcomes.

Most medical students agree AI will advance in medical practice, rather than replace medical practitioners, and should be included in medical training [4,5].

Both optimism and pessimism exist simultaneously, in which the perspectives on AI and robotics development vary widely, as some believe they are harmful and are feared for misuse while others believe they are beneficial and seen as facilitators for societal improvement [6].

With AI becoming more prevalent, medical students are increasingly using it in their education and training. Despite that, there is a lack of studies that measure the scope of this phenomenon, causing medical faculty to worry that students may have misconceptions about the advantages and disadvantages of AI in medical education. Further research is needed to completely understand the effects of AI inclusion in medical curricula [7].

Pintos Dos Santol et al. surveyed 263 medical students from three universities in Germany regarding their attitudes toward the use of AI in the medical field, particularly radiology. It was found that 52% of individuals were knowledgeable of AI discussions in radiology, while 68% lacked information about the technologies. Many students thought AI would alter radiology and medicine dramatically and should be incorporated into medical training [4].

Healthcare professionals in Pakistan lack awareness about the role of artificial intelligence in healthcare as shown in a study [8], where further investigations are required to address their issues and bridge the gap between doctors and technology to adopt a patient-centered approach to medicine.

Sit et al. surveyed the opinions of AI in healthcare among 484 medical students from 19 United Kingdom medical schools [9]. The majority (78%) recognized the significance of AI, perceived advantages in mastering it, and believed AI should be expanded into their learning. Only a small number (<10%) have been taught AI while the majority acquired knowledge from the media. Mousavi Baigi et al. reviewed 38 research papers on the opinions of healthcare students toward AI. Findings indicated that the majority (76%) of students had favorable and promising attitudes toward AI, yet 24% were deficient in understanding AI and had a negative attitude towards engaging with AI [10].

An online 15-question semi-structured survey conducted on 121 medical students and 52 clinical faculty members revealed that AI topics in medicine were only known by 30% of students and 50% of faculty. Many students (72%) and faculty (59%) discovered AI through the media. Professors were found to have a higher probability of not possessing a fundamental grasp of AI technologies in comparison to students. Students were interested in AI for training in patient care, whereas faculty favored AI for training in teaching [11]. Future healthcare workers need to learn and familiarize themselves with AI techniques like machine learning (ML) and deep learning (DL), understand their potential uses and limitations, as well as be able to identify inaccuracies in AI algorithms [12].

The recent conference hosted and organized by the College of Medicine and Health Sciences at the Arabian Gulf University entitled (Towards Future Doctors: Innovations and Prospects) has highlighted the urgent need to explore the current and future opportunities in the utilization of AI in health profession education.

## Materials and methods

Aim of the study

To explore the attitudes and knowledge of AI among medical students and its involvement in health professions education.

Study design

A descriptive cross-sectional study design was employed in this study.

Study population

All medical students (male and female; medical years one to six) enrolled at the Arabian Gulf University during the academic year 2023-2024 were included in this study.

Study instruments

A self-administered questionnaire was developed to explore the familiarity and perception of medical students regarding AI implications in medical curricula (Appendices). Students were strongly encouraged via three reminders to fill in and submit their responses to the questionnaire.

Data collection

Information was collected, coded, and entered into a micro-computer program based on the responses received from the students.

Statistical analysis

Statistical analysis was conducted using the Statistical Package for Social Sciences (SPSS) version 29 (IBM Corp., Armonk, NY, US) while the internal consistency and reliability questionnaire was measured by Cronbach’s alpha. Categorical variables were presented as frequencies and percentages, whereas continuous variables were presented as mean and standard deviation (SD). Categorical variables were compared using a chi-square test. A p-value of <0.05 was considered statistically significant.

Ethical considerations

This study was approved by the Research and Ethics Committees of the CMHS at AGU (approval number: E38-PI-3-23). The names of students chosen to answer the questionnaire were kept anonymous. All data were kept confidential.

## Results

Table 1 shows the socio-demographic characteristics of 152 medical students in the sample, with 41 (27%) males and 111 (73%) females. Students were divided into premedical (n=28, 18.4%), preclinical (n=72, 47.4%), and clinical (n=52, 34.2%) phases. Most were aged 20-24 (n=99, 69.7%) while 27 (19%) were under 20 and 16 (11.3%) were over 25. The majority were Bahraini (n=58, 38.2%), followed by Kuwaiti (n=49, 32.2%), Omani (n=27, 17.8%), Saudi (n=16, 10.5%), and other nationalities (n=2, 1.3%).

**Table 1:** Socio-demographic characteristics of medical students (n=152)

	Frequency (Percentage, %)
Gender	
Male	41 (27)
Female	111 (73)
Medical Year	
Pre-medical phase	28 (18.4)
Pre-clinical phase	72 (47.4)
Clinical phase	52 (34.2)
Age Group (Mean ± SD=21.96±2.23)	
< 20 Years	27 (19)
20-24 Years	99 (69.7)
>=25 Years	16 (11.3)
Nationality	
Bahraini	58 (38.2)
Saudi	16 (10.5)
Kuwaiti	49 (32.2)
Omani	27 (17.8)
Other	2 (1.3)

Table 2 shows that 41 (27%) students were very familiar with AI, with slightly different rates across phases of study. Most students (n=92, 60.5%) were somewhat familiar while 19 students (12.5%) were not familiar with AI. A significant difference in AI familiarity existed across the educational phases (p-value = 0.031). Only 12 (7.9%) were very familiar with AI applications in medicine. Most students (n=88, 57.9%) were somewhat familiar while 52 students (34.2%) were not familiar. There was no significant difference in AI application familiarity across phases (p-value = 0.339). Moreover, 41 (27%) students had been exposed to AI in their studies, with higher exposure in the clinical phase. However, 111 students (73%) reported no exposure, and there was no significant difference across phases (p-value = 0.064).

**Table 2:** Medical students’ knowledge of artificial intelligence (AI) according to their medical phase *: statistically significant at the 0.05 level

Statement	Categories	n (%)	Medical Year - Phase n (%)			Chi-Square Value	P-Value
			Pre-medical phase	Pre-clinical phase	Clinical phase		
What is your current level of familiarity with artificial intelligence (AI)?	Very familiar	41 (27)	9 (32.1)	18 (25)	14 (26.9)	10.611	0.031*
	Somewhat familiar	92 (60.5)	16 (57.1)	39 (54.2)	37 (71.2)		
	Not at all familiar	19 (12.5)	3 (10.7)	15 (20.8)	1 (1.9)		
How familiar you are with artificial intelligence (AI) applications in medicine?	Very familiar	12 (7.9)	2 (7.1)	4 (5.6)	6 (11.5)	4.530	0.339
	Somewhat familiar	88 (57.9)	17 (60.7)	38 (52.8)	33 (63.5)		
	Not at all familiar	52 (34.2)	9 (32.1)	30 (41.7)	13 (25)		
Have you had any exposure to artificial intelligence (AI) in your medical studies?	Yes	41 (27)	5 (17.9)	16 (22.2)	20 (38.5)	5.490	0.064
	No	111 (73)	23 (82.1)	56 (77.8)	32 (61.5)		

Table 3 presents the impact of AI on medical education, where 124 students (81.6%) believed AI would positively affect medical education while 28 (18.4%) thought it would have a negative impact. The perception differed significantly across educational phases (p-value = 0.049); 26 (17.1%) students felt very comfortable using AI in their studies while 56 (36.8%) felt somewhat comfortable. Comfort levels varied significantly across phases (p-value = 0.033). Moreover, 104 students (68.4%) supported including AI in medical curricula while 48 (31.6%) opposed it. Opinions differed significantly by phase (p-value = 0.037); 40 students (26.3%) thought resources for learning AI were adequate but 112 (73.7%) believed they were lacking. A significant difference existed between phases (p-value = 0.007).

**Table 3:** Medical students’ perception regarding artificial intelligence (AI) according to their medical phase *: statistically significant at the 0.05 level

Statement	Categories	n (%)	Medical Year - Phase n (%)			Chi-Square Value	P- Value
			Pre-medical phase	Pre-clinical phase	Clinical phase		
How do you think artificial intelligence (AI) will impact the future of medical education?	It will have a positive impact	124 (81.6)	24 (85.7)	53 (73.6)	47 (90.4)	6.043	0.049*
	It will have a negative impact	28 (18.4)	4 (14.3)	19 (26.4)	5 (9.6)		
How do you feel about using artificial intelligence (AI) in medical studies?	Very comfortable	26 (17.1)	2 (7.1)	11 (15.3)	13 (25)	16.698	0.033*
	Somewhat comfortable	56 (36.8)	16 (57.1)	20 (27.8)	20 (38.5)		
	Neutral	51 (33.6)	10 (35.7)	30 (41.7)	11 (21.2)		
	Somewhat uncomfortable	16 (10.5)	0 (0)	9 (12.5)	7 (13.5)		
	Very uncomfortable	3 (2)	0 (0)	2 (2.8)	1(1.9)		
Do you think artificial intelligence (AI) has the potential to replace teachers/doctors in medical education?	Yes	48 (31.6)	10 (35.7)	23 (31.9)	15 (28.8)	0.406	0.816
	No	104 (68.4)	18 (64.3)	49 (68.1)	37 (71.2)		
Do you think that medical schools should use artificial intelligence (AI) in their curricula?	Yes	104 (68.4)	21 (75)	42 (58.3)	41 (78.8)	6.568	0.037*
	No	48 (31.6)	7 (25)	30 (41.7)	11 (21.2)		
Do you think that medical schools should teach artificial intelligence (AI) applications to medical students?	Yes	117 (77)	24 (85.7)	51 (70.8)	42 (80.8)	3.161	0.206
	No	35 (23)	4 (14.3)	21 (29.2)	10 (19.2)		
Do you think that resources are adequately available for medical students to learn about artificial intelligence (AI) and its applications in medicine?	Yes	40 (26.3)	14 (50)	15 (20.8)	11 (21.2)	9.931	0.007*
	No	112 (73.7)	14 (50)	57 (79.2)	41 (78.8)		
Do you trust artificial intelligence (AI) applications in your medical studies?	Yes	51 (33.6)	8 (28.6)	21 (29.2)	22 (42.3)	2.721	0.257
	No	101 (66.4)	20 (71.4)	51 (70.8)	30 (57.7)		
Do you see any ethical issues in using artificial intelligence (AI) applications in medical studies?	Yes	29 (19.2)	7 (25)	15 (21.1)	7 (13.5)	1.880	0.391
	No	122 (80.8)	21 (75)	56 (78.9)	45 (86.5)		

Forty-four percent (n=67) of medical students had used AI applications, with usage varying across educational phases, though there is no significant difference (p = 0.110). Only 47 students (30.9%) had used AI in their studies, again with no significant difference (p = 0.189). Additionally, 80 students (52.6%) would recommend AI applications, with no phase-based variation (p = 0.081) (Table 4).

**Table 4:** Medical students’ practices in artificial intelligence (AI) according to their medical phase

Statement	Categories	n (%)	Medical Year - Phase n (%)			Chi-Square Value	P-Value
			Pre-medical phase	Pre-clinical phase	Clinical phase		
In general, have you ever used artificial intelligence (AI) applications?	Yes	67 (44.1)	17 (60.7)	27 (37.5)	23 (44.2)	4.408	0.110
	No	85 (55.9)	11 (39.3)	45 (62.5)	29 (55.8)		
In your medical studies, have you ever used artificial intelligence (AI) applications?	Yes	47 (30.9)	7 (25)	19 (26.4)	21 (40.4)	3.332	0.189
	No	105 (69.1)	21 (75)	53 (73.6)	31 (59.6)		
Would you recommend artificial intelligence (AI) applications to other students?	Yes	80 (52.6)	17 (60.7)	31 (43.1)	32 (61.5)	5.037	0.081
	No	72 (47.4)	11 (39.3)	41 (56.9)	20 (38.5)		

Table 5 presents the relationships between medical student (n=152) characteristics and their educational phase. Male students were 25-32.1% and females were 67.9-75% across all phases, with no significant difference (p = 0.770). Younger students were more common in the early phases while older students were primarily in the clinical phase, showing a significant difference (p < 0.01). Bahraini and Kuwaiti students increased in representation in later phases while Saudi and Omani student percentages declined, also showing a significant difference (p < 0.01).

**Table 5:** Relationship between characteristics of medical students and their medical phase *: statistically significant at the 0.05 level

Characteristics	Categories	Medical Year - Phase n (%)			Chi-Square Value	P-Value
		Pre-medical phase (n=27)	Pre-clinical phase (n=72)	Clinical phase (n=52)		
Gender	Male	9 (32.1)	18 (25)	14 (26.9)	0.522	0.770
	Female	19 (67.9)	54 (75)	38 (73.1)		
Age group	< 20 Years	19 (76)	8 (12.3)	0 (0)	92.323	<0.001
	20-24 Years	6 (24)	57 (87.7)	36 (69.2)		
	>=25 Years	0 (0)	0 (0)	16 (30.8)		
Nationality	Bahraini	10 (35.7)	27 (37.5)	21 (40.4)	23.297	0.003*
	Saudi	8 (28.6)	4 (5.6)	4 (7.7)		
	Kuwaiti	3 (10.7)	24 (33.3)	22 (42.3)		
	Omani	6 (21.4)	17 (23.6)	4 (7.7)		
	Other	1 (3.6)	0 (0)	1 (1.9)		

**Figure 1:**
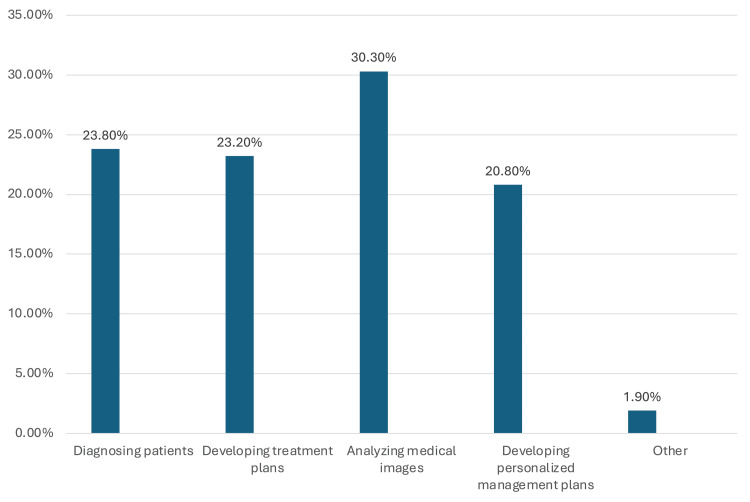
Percentage of medical students' responses related to the benefits of artificial intelligence (AI) in the medical field

Figure 1 indicates that 46 medical students (30.3%) used AI to analyze medical images, 36 (23.8%) for diagnosing patients, 35 (23.2%) for developing treatment plans, 32 (20.8%) for personalized management plans, and n=3 (1.9%) for other purposes in the medical field.

**Figure 2:**
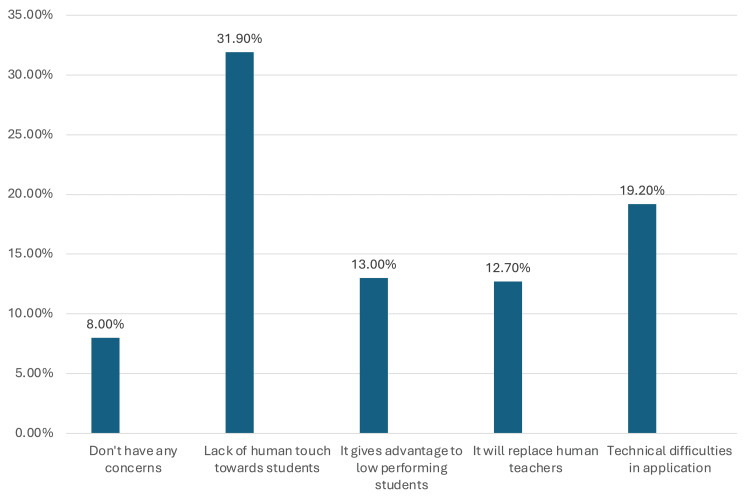
Percentages of medical student's responses related to concerns of using artificial intelligence (AI) in medical education

Figure 2 presents the potential concerns of medical students regarding AI in medical education including: 48 (31.9%) worried about the lack of human interaction, 29 (19.2%) about technical difficulties, 20 (13%) believed AI benefits low-performing students, 19 (12.7%) feared AI might replace human teachers, and 12 (8%) had no concerns at all.

## Discussion

Our study explored the awareness, perceptions, and familiarity of Gulf Arab medical students with artificial intelligence during the different phases of their medical education. Twenty-seven percent (n=41) of the medical students said that they were very familiar with artificial intelligence applications while 92 (60.5%) of students reported that they were somewhat familiar with AI. Interestingly, the level of familiarity of medical students with AI applications was significantly higher as the students progressed in their medical education phases, with senior medical students in the clinical phase of the medical program being significantly (p = 0.031) more familiar with AI applications compared to their junior colleagues. However, there was no significant difference among medical phases in student’s perceptions regarding the application of AI in medical practice (p=0.339).

The familiarity and exposure of medical students to research methodology and research studies appear to significantly increase their familiarity with AI applications (p=0.033). This may reflect the increasingly wide use of AI programs among medical students in writing and developing research protocols as well as the final research reports. Most medical students (n=124, 81.6%) believed that AI would positively impact medical education. However, their perceptions varied significantly by educational phase (p=0.049). Similarly, it was found that the majority of students (n=104, 68.4%) strongly advocated integrating AI into medical curricula, citing insufficient resources (n=112, 73.7%) as a potential barrier. Medical students also raised justifiable concerns regarding the wide use of AI in medical education and practice, particularly regarding the impact of AI on reducing human touch in medical practice and doctor-patient communication (n=48, 31.9%). In addition, the medical students raised concerns regarding the technical challenges that students face when applying AI (n=29, 19.2%).

One of the main strengths of this study is that it was conducted in a medical school that hosts a diverse population of medical students from different Arab Gulf countries; including both male and female students. In addition, the study comprises students from different phases of the medical education curriculum (pre-medical, pre-clinical, and clinical). Thus, the study results provide a comprehensive description of the familiarity and perception of medical students with AI, which can be extrapolated to other medical institutes. Further, the detailed demographic breakdown of the data is highly relevant in developing and implementing future policies in medical education.

In this study, it was found that 12 (7.9%) medical students were very familiar with AI applications in medicine, 88 (57.9%) were somewhat familiar, and n=52 (34.2%) were not familiar with AI applications in medicine. This represents an increasing familiarity of medical students with AI applications compared to previously published literature. For example, a European study conducted in 2019 reported that 52% of students were aware of the application of AI in radiological diagnosis while 68% were not aware of the involvement of AI technologies in medicine [4]. This trend of increasing acceptability of medical students for applying AI in medical practice is supported by the findings of our study with only 28 (18.4%) medical students not in favor of the use of AI within medical curricula. A recent study from Kuwait revealed that most students understood the basic principles of AI [5]. Further, another study reported that students thought that the implementation of AI technologies in medical curricula was inevitable [6]. Similarly, a study from Pakistan found that around 68.8% of medical students were aware of AI applications in medicine [7]. This study has shown that most (n=100, 65.8%) medical students were at least somewhat familiar with AI applications in medicine. However, in contrast, it seems that, unlike the situation with medical students, a substantial proportion of doctors were not aware of the basic concepts of AI and its potential applications in medicine [4,13-16].

Some shortcomings of this study should be considered before generalizing the findings. The design of the study was cross-sectional, which only provides insight into the familiarity and perceptions of AI among medical students at a single point in time. Thus, such a design does not provide the opportunity to monitor the changes in the perceptions of medical students of AI over long periods of time in an era of fast and rapid evolution in the integration of AI technologies into medical education and clinical practice. Nevertheless, we believe that the wide diversity of the medical students enrolled in this study along with the directions provided by the sub-analysis provides invaluable data for planning and developing future educational policies in the field of medical education. Thus, developing educational implications should be researched further, as a systemic study that was conducted suggests that if any country wants to develop and use AI, training should be provided first [10]. Also, faculty members should be researched regarding awareness of the uses of AI to develop AI use in medical education as discussed in research that faculty have an encouraging interest in AI training [11].

## Conclusions

Arab Gulf medical students generally demonstrated positive attitudes and familiarity with the applications of AI in medical education. Tailored educational strategies are needed to optimize AI integration in medical practice and address current concerns about AI in the medical community.

## Appendices

Questionnaire

1.    Gender

o   Male

o   Female

2.    Year of Birth: _______

3.    Nationality:

o   Bahrain

o   Saudi

o   Kuwaiti

o   Omani

o   Emirati

o   Qatari

o   Other: Specify: _________________

4.    Medical Year:

o   Foundation Year

o   Year 1

o   Year 2

o   Year 3

o   Year 4

o   Year 5

o   Year 6

5.    What is your current level of familiarity with artificial intelligence (AI)?

o       Very familiar

o       Somewhat familiar

o       Not at all familiar

6.    How familiar you are with artificial intelligence (AI) applications in medicine?

o       Very familiar

o       Somewhat familiar

o       Not at all familiar

7.    To your knowledge, have you had any exposure to artificial intelligence (AI) in your medical studies?

o       Yes

o       No

o       I'm not sure           

8.    Which of the following areas do you think artificial intelligence (AI) could be most useful in medical education? (You may select more than one answer)

o       Diagnosing patients

o       Developing treatment plans

o       Analyzing medical images

o       Providing personalized learning experiences

o       Other (please specify): ___________

9.    How do you think artificial intelligence (AI) will impact the future of medical education?

o       It will have a positive impact

o       It will have a negative impact

o       It will have no impact

o       I'm not sure

10. Do you think artificial intelligence (AI) has the potential to replace teachers/doctors in medical education?

o       Yes

o       No

o       I'm not sure

o       It depends on the subject/specialty (explain your answer): ___________

11. How do you feel about using artificial intelligence (AI) in medical education?

o       Very comfortable

o       Somewhat comfortable

o       Neutral

o       Somewhat uncomfortable

o       Very uncomfortable

12. What concerns do you have about the use of artificial intelligence (AI) in medical education? (You may select more than one answer)

o   I don’t have any concerns   

o   Lack of human touch towards students

o   It gives advantage to low performance students

o   Replacement of human teachers

o   Technical difficulties in applications

o   Other (please specify): ______________

13. Do you think that medical schools should use artificial intelligence (AI) in their curricula?

o       Yes

o       No

o       I'm not sure

o       It depends on the subject/specialty (explain your answer): ___________

14. Do you think that medical schools should teach artificial intelligence (AI) applications to medical students?

o       Yes

o       No

o       I'm not sure

o       It depends on the subject/specialty (explain your answer): ___________

15. Do you think that resources are adequately available for medical students to learn more about artificial intelligence (AI) and its applications in medicine?

o       Yes

o       No

o       I'm not sure

o       It depends on the subject/specialty (explain your answer): ___________

16. In general, have you ever used artificial intelligence (AI) applications?

o       Yes

o       No

o       I'm not sure

17. In your medical studies, have you ever used artificial intelligence (AI) applications?

o       Yes

o       No

o       I'm not sure

18. Do you trust artificial intelligence (AI) applications in medical studies?

o       Yes

o       No

o       I'm not sure

19. Would you recommend artificial intelligence (AI) applications to other students?

o       Yes

o       No

o       I'm not sure

20. In your opinion, do you see any ethical issues in using artificial intelligence (AI) applications in medical studies?

o   Yes:  Please specify: _______

o   No

o   I'm not sure

Thank you for taking the time to complete this questionnaire!

## References

[1] Chen M, Zhang B, Cai Z (2022). Acceptance of clinical artificial intelligence among physicians and medical students: a systematic review with cross-sectional survey. Front Med (Lausanne).

[2] (2024). What is artificial intelligence (AI)?. https://www.ibm.com/topics/artificial-intelligence.

[3] Garcia MB, Arif YM, Khlaif ZN, Zhu M, de Almeida RPP, de Almeida RS, Masters K (2024). Effective integration of artificial intelligence in medical education: practical tips and actionable insights. Transformative Approaches to Patient Literacy and Healthcare Innovation.

[4] Pinto Dos Santos D, Giese D, Brodehl S (2019). Medical students' attitude towards artificial intelligence: a multicentre survey. Eur Radiol.

[5] Buabbas AJ, Miskin B, Alnaqi AA, Ayed AK, Shehab AA, Syed-Abdul S, Uddin M (2023). Investigating students’ perceptions towards artificial intelligence in medical education. Healthcare (Basel).

[6] Neudert L-M, Knuutila A, Howard PN (2020). Global attitudes towards AI, machine learning & automated decision making. Oxford Commission on AI & Good Governance. https://oxcaigg.oii.ox.ac.uk/publications/global-attitudes-towards-ai-machine-learning-automated-decision-making-2/.

[7] Gillissen A, Kochanek T, Zupanic M, Ehlers J (2022). Medical students’ perceptions towards digitization and artificial intelligence: a mixed-methods study. Healthcare (Basel).

[8] Ahmed Z, Bhinder KK, Tariq A (2022). Knowledge, attitude, and practice of artificial intelligence among doctors and medical students in Pakistan: a cross-sectional online survey. Ann Med Surg (Lond).

[9] Sit C, Srinivasan R, Amlani A, Muthuswamy K, Azam A, Monzon L, Poon DS (2020). Attitudes and perceptions of UK medical students towards artificial intelligence and radiology: a multicentre survey. Insights Imaging.

[10] Mousavi Baigi SF, Sarbaz M, Ghaddaripouri K, Ghaddaripouri M, Mousavi AS, Kimiafar K (2023). Attitudes, knowledge, and skills towards artificial intelligence among healthcare students: a systematic review. Health Sci Rep.

[11] Wood EA, Ange BL, Miller DD (2021). Are we ready to integrate artificial intelligence literacy into medical school curriculum: students and faculty survey. J Med Educ Curric Dev.

[12] Sapci AH, Sapci HA (2020). Artificial intelligence education and tools for medical and health informatics students: systematic review. JMIR Med Educ.

[13] Aldakhil S, Alkhurayji K, Albarrak S (2024). Awareness and approaches regarding artificial intelligence in dentistry: a scoping review. Cureus.

[14] Dashti M, Londono J, Ghasemi S (2024). Attitudes, knowledge, and perceptions of dentists and dental students toward artificial intelligence: a systematic review. J Taibah Univ Med Sci.

[15] Jebreen K, Radwan E, Kammoun-Rebai W (2024). Perceptions of undergraduate medical students on artificial intelligence in medicine: mixed-methods survey study from Palestine. BMC Med Educ.

[16] Al Hadithy ZA, Al Lawati A, Al-Zadjali R, Al Sinawi H (2023). Knowledge, attitudes, and perceptions of artificial intelligence in healthcare among medical students at Sultan Qaboos University. Cureus.

